# Supplementary material for: Coherence in carotenoid-to-chlorophyll energy transfer
Source: Nat Commun. 2018 Aug 8;9:3160. doi: 10.1038/s41467-018-05596-5 (PMC6082895; doi:10.1038/s41467-018-05596-5)
Supplement: Supplementary file 1 — Supplementary Information [file 41467_2018_5596_MOESM1_ESM.pdf]

Coherence in carotenoid-to-chlorophyll energy transfer

Supplementary Information

Meneghin *et al.*

Supplementary Methods 1: Quantum Chemical Methods

Exciton model. The excited states of the whole PCP system are described through an exciton model. The exciton Hamiltonian $\hat{H}_{ex}$ for a system of N pigments, each bearing n_i excitations, reads:

$$\hat{H}_{ex} = \sum_{i}^{N}\sum_{a}^{n_i} \mathcal{E}_i^a \left|ia\right\rangle \left\langle ia\right| + \sum_{ij}^{N}\sum_{ab}^{n_i n_j} V_{ij}^{ab} \left|ia\right\rangle \left\langle jb\right| \tag{S1}$$

where $\mathcal{E}_i^a$ is the a-th excitation energy of pigment i (site energy), V_{ij}^{ab} is the electronic coupling between two transitions of different pigments. Diagonalization of the Hamiltonian matrix in Eq. S1 yields the energy levels of the exciton states E_K and the exciton coefficients C_{ia}^K. The excitonic Hamiltonian of a PCP monomer includes the S_2 bright states of the eight Pers, and the Q and Soret (B) bands of the two chlorophylls (Chls), resulting in 16 total states.

Spectral densities of the exciton-phonon coupling. The spectral densities (SD) of the different pigments and transitions were modeled as a sum of one cubic continuous contribution and M discrete contributions:

$$C_{ia}(\omega) = \frac{1}{2}\lambda_{c,ia}\left(\frac{\omega}{\omega_c}\right)^3 e^{-\frac{\omega}{\omega_c}} + \sum_{k=0}^{M} S_{k,ia}\,\omega_{k,i}\,\frac{\omega\gamma_{k,i}}{(\omega-\omega_{k,i})^2+\gamma^2} \tag{S2}$$

where i and a indicate the pigment and transition of interest, respectively, k runs on the vibrational modes of the pigment, λ_c is the continuous contribution to the reorganization energy, and ω_c is the cutoff frequency of the continuous contribution. Finally, $\omega_{k,i}$ and $S_{k,ia}$ are the frequency and Huang-Rhys factor of mode k in pigment i / transition a. The total reorganization energy is then

$$\lambda_{ia} = \int_0^\infty \frac{C_{ia}(\omega)}{\pi\omega}\mathrm{d}\omega = \lambda_{c,ia} + \sum_{k=0}^{M} S_{k,ia}\,\omega_{k,i} \tag{S3}$$

The discrete part of the SD was determined from a normal mode analysis [1] of the pigments in the crystal structure. The Huang-Rhys factor $S_{k,j}$ of each mode k was computed by projecting the gradient of the excited state j on the ground-state normal mode k [1]. The computed spectral densities are shown in Supplementary Fig. 1.

Spectra and rates. The exciton spectra of PCP were simulated employing the disordered exciton model coupled to the modified Redfield theory (mR) [2–4] for the description of exciton relaxation. The static disorder was modeled by allowing random Gaussian uncorrelated fluctuations of the site energies (parameters in Supplementary Table 1). For each realization of the disorder, the

Hamiltonian in Eq. S1 is diagonalized, obtaining exciton energies E_K and exciton coefficients C_j^K for every state $|K\rangle$. The spectrum is then calculated as:

$$A(\omega) \propto 2\omega \sum_K |\boldsymbol{\mu}_{0K}|^2 \Re \int_0^\infty \mathrm{d}t \, e^{i(\omega-\omega_K)t - g_K(t) - \frac{t}{2\tau_K}} \tag{S4}$$

where $\omega_K = E_K/\hbar$ is the vertical excitation frequency of state K, τ_K is the exciton lifetime as computed with mR theory, $g_K(t)$ represents the lineshape function of exciton state K,

$$g_K(t) = -\int_0^\infty \frac{C_{KKKK}(\omega)}{\pi\omega^2} \left[\coth\left(\frac{\beta\hbar\omega}{2}\right) \cdot \big(\cos(\omega t) - 1\big) \; - \; i\,(\sin(\omega t) - \omega t)\right] \tag{S5}$$

and $C_{KKKK}(\omega)$ is the spectral density of exciton state K, which depends on the exciton coefficients and the spectral density of each state ia.

$$C_{KLMN}(\omega) = \sum_j c_{ia}^K c_{ia}^L c_{ia}^M c_{ia}^L C_{ia}(\omega) \tag{S6}$$

Computational details. All calculations are based on the crystal structure of main-form PCP (Ref. [5], PDB code: 1PPR). Hydrogen atoms were added using the LeAP module of AmberTools [6], considering all the titrable residues in their standard protonation state. The crystal structure was refined by relaxing the geometries of all the pigments through a QM/MM optimization in the ONIOM framework with electrostatic embedding [7], followed by normal modes and frequency calculations. At the optimized geometry, the gradient of the excited states was also computed in order to extract Huang-Rhys factors (see below). All QM/MM optimizations were performed at the B3LYP/6-31G(d) level of theory, keeping the MM part fixed. Note that B3LYP/6-31G(d) was found to give quite reasonable results for the geometry of peridinin (Per) [8].

The parameters for the protein were taken from the Amber ff99SB forcefield [9]. The charges of the cofactors were fitted to the HF/6-31G(d) electrostatic potential using the standard RESP procedure using AmberTools [6]. Selected Pers (614 and 624) were optimized in their S_2 excited state, using the same QM/MM ONIOM scheme with time-dependent DFT (TD-DFT). Additional optimizations were performed with the CAM-B3LYP functional.

The excitation energies of each pigment were computed at TD-DFT B3LYP/6-31G(d) level. The electronic couplings among all the excited states were computed with an approach based on the transition densities of the interacting pigments [10]. For all the site energy and coupling calculations, the protein environment was described through a polarizable QM/MM embedding (QM/MMPol) [10], in which the pigments of interest are described quantum mechanically, whereas the protein and the other cofactors are treated as a set of point charges and isotropic polarizabilities. The parameters for the MMPol forcefield were taken from Wang *et al.* [11], except for the MMPol charges of the pigments,

which were fitted to the B3LYP/6-31G(d) electrostatic potential consistently with the polarization.

Supplementary Methods 2: Kinetic global analysis

The total 2DES signal recorded using laser 3 was global fitted using the kinetic model reported in Figure 5a in the main text. We are interested in the cascading signal from the excitation of the Pers, thus we selected the excitation frequency range from 1550 cm^{-1} to 18000 cm^{-1} for the analysis. The method is based on the global analysis of 2D electronic spectroscopy (2DES) data reported in Ref. [12]. In this work, we focus the analysis on the population dynamics using a suitable basis set of kinetic decay components. The objective of the analysis is to determine the time constants $T_{e'e}$ of the energy transport processes $e \to e'$, and to get spectral information on where a particular process contributes to the 2DES maps.

Kinetic model. We employed a three states kinetic model for which we can build the population transport rate matrix as

$$K_{ee'} = -\frac{1}{T_{e'e}} + \delta_{e'e} \sum_{e''} \frac{1}{T_{e''e}} \tag{S7}$$

where $e, e' = \mathrm{S_2}, \mathrm{S_1}, \mathrm{Q_y}$. Several constraints can be put into this matrix, mainly some transport channels can be switched off. The specific transport rate matrix can be specified as

$$\mathbf{K} = \begin{bmatrix} 0 & 0 & 0 \\ K_{\mathrm{S_1Q_y}} & 0 & 0 \\ K_{\mathrm{S_2Q_y}} & K_{\mathrm{S_2S_1}} & 0 \end{bmatrix}, \tag{S8}$$

where zero elements stand for switched off channels. Using matrix $\mathbf{K}$ we can calculate the population Green's functions [13], organized in matrix $\mathbf{G}(t)$ and we can solve the Pauli master equation

$$\frac{d\mathbf{G}(t)}{dt} = -\mathbf{K}\mathbf{G}(t). \tag{S9}$$

Each population Green's function $G_{e'e}(t)$ expresses the probability of the population of state e to be transferred to state e' at time t. We selected only the diagonal components $G_{ee}(t)$ to be used in the fitting procedure as they represent a linearly independent basis set for all the population Green's functions. In order to account for the inhomogeneity of the population transfer channel $\mathrm{S_2} \to \mathrm{Q_y}$ the population Green's functions were averaged for 500 realizations of the kinetics problem using a lognormal distributed time constant $T_{\mathrm{Q_yS_2}}$. The choice of the lognormal probability distribution shape is guided by the satisfactorily match with the computed rates histogram reported in Supplementary

Fig. 5 and by the limited number of its parameters. The population Green's functions of the basis set are stored as column vectors in matrix C_{kn}, where n indexes the n-th kinetic component. Each time the matrix $\mathbf{C}(\mathbf{K})$ is evaluated the full averaged kinetic problem is solved.

Global fitting. The experimental data is represented as a three dimensional array X_{ijk}, where i and j are the indexes of emission and excitation frequencies and k is the index the population time t. We modeled the data as a sum of separated contributions expressed as the product of complex amplitude maps, stored as pages in a three dimensional array $\mathbf{A}$, and kinetic decays stored as columns of matrix $\mathbf{C}(\mathbf{K})$. Therefore, the model function $\mathbf{M}$ can be defined as

$$M_{ijk}(\mathbf{A}, \mathbf{K}) = \sum_n A_{ijn}\ C_{kn}(\mathbf{K}). \tag{S10}$$

The problem is written in term of the unconstrained minimization of the least squares of the residuals, $\mathbf{R}(\mathbf{A}, \mathbf{K}) = \mathbf{X} - \mathbf{M}(\mathbf{A}, \mathbf{K})$, obtaining

$$\min_{\mathbf{A},\mathbf{K}} \|\mathbf{R}(\mathbf{A}, \mathbf{K})\|_2^2. \tag{S11}$$

The minimization over the complex amplitudes is analytically solved employing the variable projection algorithm [12,14]. In this way the residuals are specified as a function of matrix $\mathbf{K}$ only as

$$\tilde{R}_{ijk}(\mathbf{K}) = \sum_{k',n} \left(I_{kk'} - C_{kn}(\mathbf{K})C^{+}_{nk'}(\mathbf{K})\right) X_{ijk'} \tag{S12}$$

where $\mathbf{I}$ is the identity matrix and $\mathbf{C}^{+}$ is the Moore-Penrose pseudo inverse of matrix $\mathbf{C}$. The simplified minimization problem becomes

$$\min_{\mathbf{K}} \|\tilde{\mathbf{R}}(\mathbf{K})\|_2^2. \tag{S13}$$

After solving the minimization problem, the complex amplitudes are reconstructed as

$$A_{ijn} = \sum_k C^{+}_{nk} X_{ijk}. \tag{S14}$$

The amplitudes of the components of the basis set of the kinetic model resulting from the global fit analysis of the pure absorptive dataset recorded with laser 3 are reported in Supplementary Fig. 6a-c. The most significant feature is represented in $G_{S_2S_2}$ map, where, on the bottom, a negative signal represents the rising behavior of (17500,14850) cm^{-1} cross-peak due to $\mathrm{S_2} \rightarrow \mathrm{Q_y}$ EET. The process is simultaneous with the decay of $\mathrm{S_2}$ ESA signal, recognized as a negative peak close to the diagonal, and with $\mathrm{S_1}$ ESA formation, as the positive feature. The decaying behavior of $\mathrm{S_1}$ ESA signal, due to EET towards $\mathrm{Q_y}$, is clearly represented by the negative signal in $G_{S_1S_1}$ map. Finally, the positive cross-peak in $G_{Q_yQ_y}$ map witnesses the formation of the $\mathrm{Q_y}$ population.

The evolution of the Green's function of the basis set components and of the significant off-diagonal elements of the kinetic model is shown in Supplementary Fig. 6d and e, respectively.

As direct consequence of the EET rates determined by the fitting analysis, >67% of initially prepared S_2 population is directly transferred to Q_y. Thus, according to our results, only a minor fraction of the Per624 population is funneled to Q_y *via* the S_1 state, as shown in Figure 5b (main text).

Supplementary Note 1: Calculated parameters

Site energies. The calculated site energies of Per and Chl a are reported in Supplementary Table 2. The calculated Q_y site energies of the two Chl in PCP are not identical, with Chl602 being ~170 cm^{-1} blue-shifted with respect to Chl601. The Q_y site energies are ~1750 cm^{-1} higher than the experimental values; however, the relative site energy difference between the two Chls is sensible, and in agreement with experimental steady-state measurements [15].

The site energies of Pers differ by more than 1000 cm^{-1}, due to the different protein environment. Surprisingly, Per624 is strongly red-shifted by the protein environment, whereas its pseudo-symmetric Per614 is among the less red-shifted. Per614 and 624 are equivalent by position and are the most interacting with Chl. However, their binding pocket is not the same. To verify that the red-shift originates from the direct effect of the protein environment, we used the internal geometry of Per614, but in the pocket of Per624, obtaining exactly the same ~1900 cm^{-1} solvatochromic shift (with respect to gas phase) observed for Per624. This demonstrates that the solvatochromic shift is not a result of the different ground-state geometry, but rather a direct electrostatic/polarization effect.

The differences between the binding pockets of the two Pers are summarized in Supplementary Fig. 2. As shown in the Figure, there are significant substitutions in the charged residues of the two pockets. In particular, there are various negatively charged residues on the allene side of Per624, which are substituted by neutral residues in Per614's pocket. On its lactone side, Per614 is hydrogen bonded to Glu101, which is substituted by Lys263 in Per624's binding pocket. The particular arrangement of charged residues around Per624 generates an electrostatic field along the Per backbone, as shown by the electrostatic potential maps of Supplementary Fig. 3 and Figure 2b in the main text. The other red-shifted Pers are 611/621 and 623. These red-shifted Pers are more likely to be excited by the laser tuned on the red tail of the Per absorption.

Exciton couplings. Supplementary Table 3–5 summarize the electronic couplings calculated in the protein environment among the pigments of PCP. The magnitude of the interactions shows that the two Chls are very weakly coupled. On the contrary, Pers are strongly coupled, with some couplings reaching values larger than 350 cm^{-1}.

Finally, turning to the Per/Chl interactions, in Supplementary Table 5 we compared the couplings between the Pers' S_2 states and the $Q_{x/y}$ states of Chl. While the couplings to Q_x states are always <100 cm^{-1}, the couplings to Q_y reach values around 300 cm^{-1}, that is same magnitude as Per-Per interactions. The differences between S_2/Q_x and S_2/Q_y couplings can be ascribed to the lower dipole strength of the Q_x states compared to the Q_y. Notably, Pers 614 and 624, which was identified as the ones that strongly interact with the Chl of the same cluster, show the largest coupling to the Q_y state.

Supplementary Note 2: Prediction of the excitonic spectrum and rate analysis

We used the exciton Hamiltonian presented in Supplementary Tables 2–5 and we calculated the absorption spectrum of PCP in the entire VIS region using modified Redfield theory. We shifted all the site energies of the Chl Q_y and B transitions by -1750 cm^{-1} (maintaining the same relative differences as calculated with QM/MMPol) to compute the whole spectrum since our QM level of theory yields different errors on the excitation energies of different transitions. In the same way, we shifted the Q_x site energies by -2100 cm^{-1}, and the S_2 energies by 3000 cm^{-1}. This gives a reasonable agreement with the experimental spectrum (see Figure 1 in the main text).

The mR simulations were also used to analyze $S_2 \rightarrow Q_y$ (and $S_2 \rightarrow Q_x$) EET rates as a function of the Per energy, based on 10 000 realizations of the static disorder. First of all we show in Supplementary Fig. 4a and b the relationship between the Per zero-phonon energy (*i.e.* the adiabatic energy $E_K - \lambda_K$) and the dipole strength and delocalization length, the latter defined as inverse participation ratio (IPR). The IPR of a Per state K is calculated on the basis of exciton coefficients in a particular realization:

$$\mathrm{IPR}_K = \left[\sum_i^{(\mathrm{Pers})} \left(c_{ia}^K \right)^4 \right]^{-1} \qquad a = \mathrm{S}_2 \tag{S15}$$

This quantity ranges from a minimum of 1, for the states localized on only one Per, to a maximum of 8, that is the total number of Pers. At the red end of the S_2 band (ZPE < 17500 cm^{-1}), the static disorder localizes the exciton on only one Per (Supplementary Fig. 4b), and the dipole strength of the excited state resembles that of a single Per (Supplementary Fig. 4a). For this reason, we can conclude that the Per states are virtually localized when exciting at the red edge of the S_2 band.

Figure 4c and d shows the relationship between zero-phonon Per energies and the $S_2 \rightarrow Q_y$ and $S_2 \rightarrow Q_x$ EET rates. When the states are localized on only one site ($c_{ia}^K > 0.9$), the rates can be assigned to one specific Per. Each Per transfers the excitation energy with a different rate depending on the coupling to the $Q_{x/y}$ state. Notably, $S_2 \rightarrow Q_x$ EET rates reach at most 3 ps^{-1}, whereas

$S_2 \rightarrow Q_y$ EET rates are much faster, between 5 and 20 ps^{-1}, which corresponds to transfer times of 50–200 fs. The largest EET rates occur at the red-most edge of the S_2 band, where the Per states are localized and the S_2/Q_y energy gap is minimum.

The Per → Chl EET essentially occurs from a single Per donor, which may be Per624, 611(621) or 613(623) depending on which is the red-most Per in a particular configuration of static disorder. Different Pers transfer the excitation to the Q state with different rates, depending on the coupling with the acceptor state. The fastest transfer to Q_y states occurs for Per624. The energy region probed in 2DES experiments corresponds to the very red edge of Per absorption, slightly above 17000 cm^{-1}. In this region, different Pers contribute to the EET toward the Q_y state, but only Per624 can transfer energy to the Q_y state of Chl with an EET time shorter than 100 fs, as observed in experiments; this Per was already proposed as the most coupled to Chl.

Supplementary Note 3: Bilinear time-frequency analysis

Signals from the S_2/Q_y cross-peaks were investigated using time-frequency methods which can provide simultaneously frequency and time resolution, unveiling the dynamics of the relevant beating components. In particular, four signals extracted from the 2DES rephasing maps obtained with laser 3 at excitation frequencies 17200, 17600, 17800, 18000 cm^{-1} and emission frequency 14850 cm^{-1} were analyzed with the smoothed-pseudo-Wigner-Ville distribution (SPWV) [16]. This time-frequency decomposition is derived from the Wigner-Ville distribution, two smoothing time windows, $h(t)$ and $g(t)$, are added in order to mitigate the presence of interference terms. SPWV is defined as

$$SPWV(t,\nu) = \int h(t') \int g(t''-t)\ s\left(t''+\frac{t'}{2}\right) s^*\left(t''-\frac{t'}{2}\right) e^{-i2\pi\nu t'} dt'dt'' \tag{S16}$$

where $s(t)$ is the investigated signal. The time windows are chosen with Gaussian shape and standard deviations $\sigma_h = 75$ fs and $\sigma_g = 100$ fs. The resulting distributions are shown in Supplementary Fig. 7. Two kinds of contributions are identified: long lasting features with time constants of hundreds of femtoseconds ascribable to Per vibrational coherences and rapidly decaying features with time constants of tens of femtoseconds assigned to electronic coherences between Q_y and S_2 states. The most striking difference between the two kinds of features is their shape in the time-frequency plane which is determined by the time-frequency uncertainty principle. Long lasting signals have a narrow frequency footprint, whereas rapidly decaying signals have a large frequency bandwidth. In the overlap between the two contributions, negative interference artifacts are present.

To clarify how the different signal contributions affect the SPWV distribution, a detailed model analysis was performed on the signal extracted at (17800,

14850) cm^{-1}. The signal was fitted with a model function composed by four oscillating components. The model function $f(t_2)$ is specified as

$$f(t_2) = \sum_{i=1}^{3} a_i exp\left(-t_2/\tau_i - i\omega_i t_2\right) + a_4 exp\left(-(t_2/\sqrt{2}\sigma_4)^2 - i\omega_4 t_2\right) \tag{S17}$$

where a_i are the complex amplitudes, τ_i are the damping times, ω_i are the oscillating frequencies and σ_4 is the standard deviation for the Gaussian damping employed for the electronic coherence. The parameters obtained from the fitting procedure are reported in Supplementary Table 6, whereas the plot of the model function, superimposed to the real data, is reported in Supplementary Fig. 8. The model fitting allowed us to calculate separately the SPWV distribution of each independent contribution to the signal. As shown in Supplementary Fig. 9, the contributions overlapping in the time-frequency plane were thus disentangled. The model fitting allowed us to calculate separately the SPWV distribution of each independent contribution to the signal and thus to disentangle the contributions overlapping in the time-frequency plane.

The time-frequency analysis has also been performed to the above diagonal Q_y/S_2 cross-peak. The interpretation of the results at those coordinates is however a bit more challenging because the overwhelming contribution of the vibrational modes of Per almost completely hide the presence of the 1900 cm^{-1} component. We report below Supplementary Fig. 10 showing the time-frequency analysis performed at (14920,17530) cm^{-1}. The presence of a broad weak quickly damped component around 2000 cm^{-1} can only be guessed. Nevertheless, there is an important indirect trace of the presence of such component, i.e., the appearance of negative artifacts.

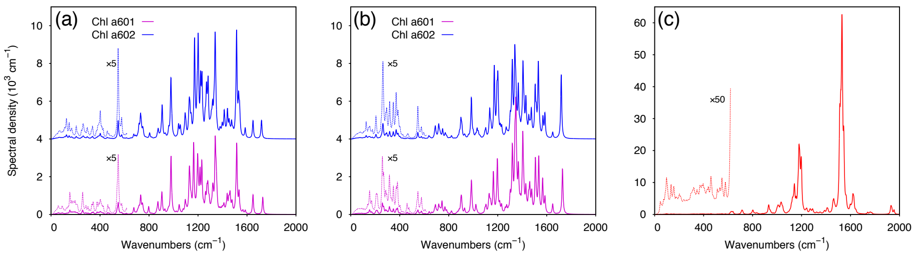

Supplementary Figure 1: Calculated spectral densities. Spectral densities for the Q_y (a) and Q_x (b) states of the two Chls, and average spectral density for the Per (c). In panels (a) and (b) the plots are offset by 4 units on the y-scale to ease the comparison. Notice the different y-axis scale in (c).

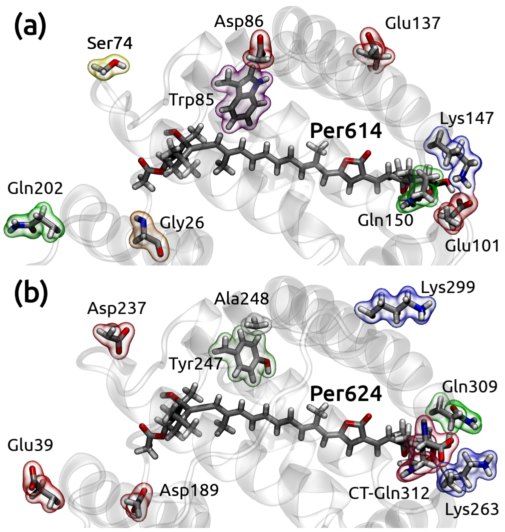

Supplementary Figure 2: Binding pockets of Per614/Per624. Representation of the differences in the binding pockets of Per614 (a) and Per624 (b). Only the polar/charged residues that differ in the two pockets are shown, and they are colored according to the residue name. Negatively and positively charged residues are highlighted in red and blue, respectively.

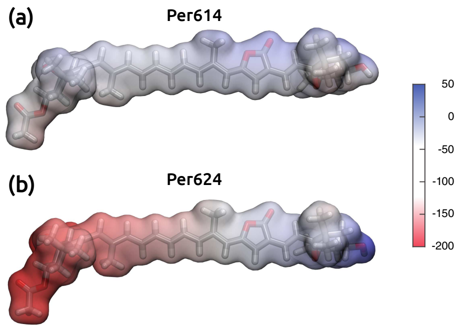

Supplementary Figure 3: Protein electrostatic potential on Per614/Per624. Electrostatic potential generated by the protein atoms plotted on the molecular surface of Per614 (a) and Per624 (b). Panel (b) is equivalent to Figure 2(b) of the main text, but with the same orientation as in Figure 2. The potential around the two Pers is plotted on the same scale.

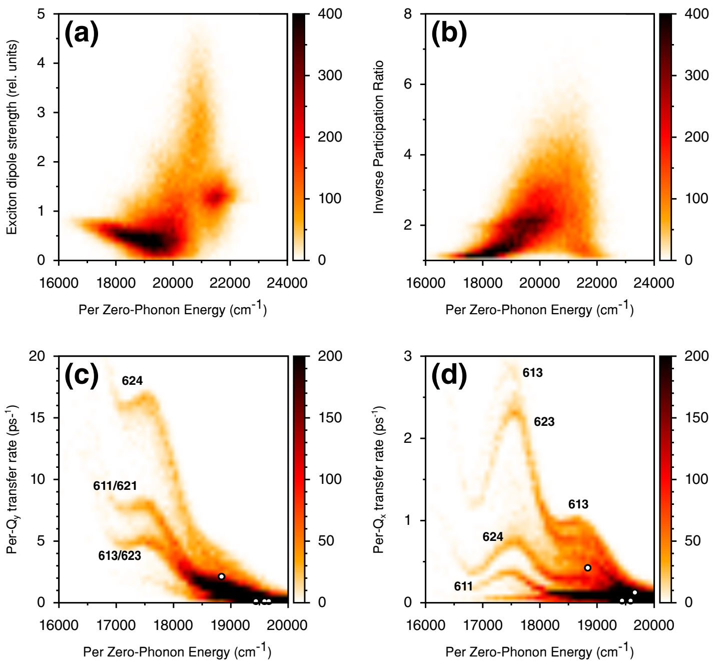

Supplementary Figure 4: Analysis of Per excitons. Heatmaps representing the zero-phonon energy of Per excitons and various quantities, such as the dipole strength (a), delocalization length (b), and rates of EET to Q_y (c) and Q_x (d) states. In panel (a) the dipole strength is normalized to that of one Per. In panels (c) and (d) the white points represent the $S_2 \rightarrow Q_{x/y}$ EET rates in the absence of disorder. The contributions from localized states are assigned to the corresponding Per. Note the different y-axis scale in panels (c) and (d).

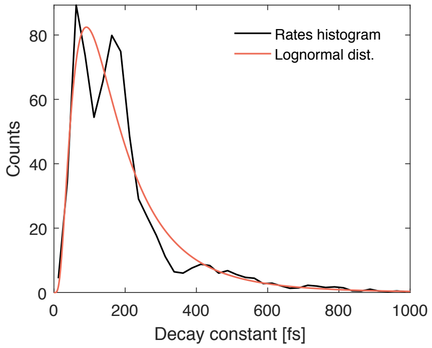

Supplementary Figure 5: Histogram of the $S_2 \rightarrow Q_y$ rates. Computed $S_2 \rightarrow Q_y$ EET rates resulting from 200 realizations of the static disorder (black) for the couple Per624/Chl602. Fitted lognormal distribution (red).

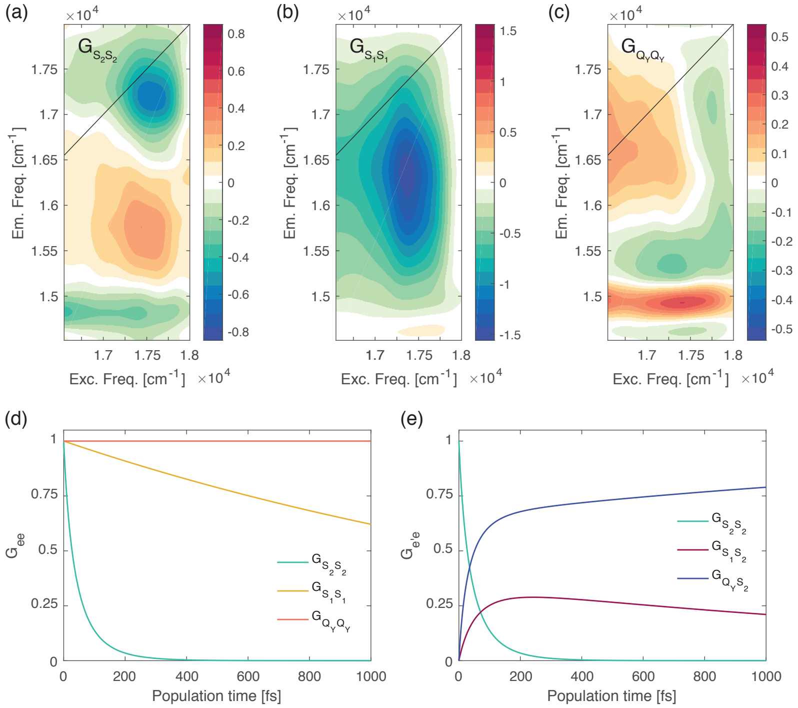

Supplementary Figure 6: Results of the fitting analysis. Fitting analysis of the pure absorptive dataset recorded with laser 3. (a-c) Real amplitude distribution of diagonal components, basis set of the kinetic model. Temporal evolution of the Green's function of diagonal (d) and of significant off-diagonal (e) components of the kinetic model.

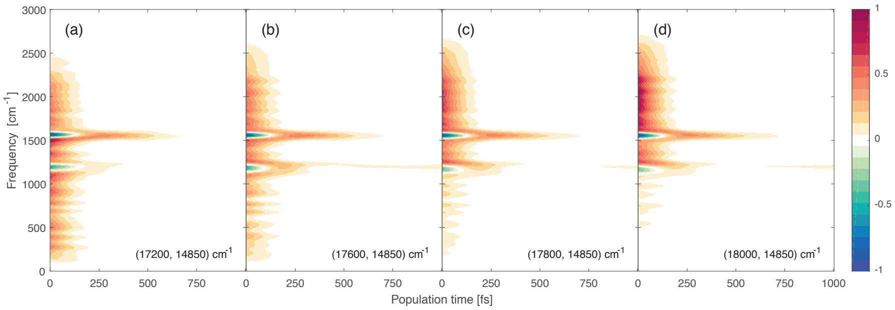

Supplementary Figure 7: Time-frequency analysis of S_2/Q_y cross-peak region. Bilinear time-frequency transform of the oscillating part of the rephasing signals recorded with laser 3 and extracted at excitation energy of 17200 cm^{-1} (a), 17600 cm^{-1} (b), 17800 cm^{-1} (c) and 18000 cm^{-1} (d).

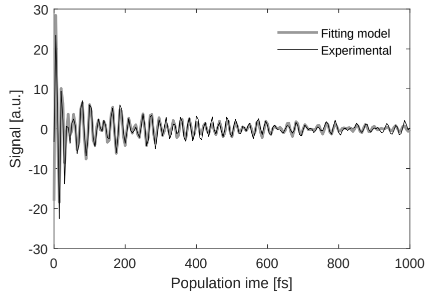

Supplementary Figure 8: Oscillating signal at S_2/Q_y position. Oscillating part of the rephasing signal recorded with laser 3 at coordinates (17800,14850) cm^{-1} (black) and fitting model function (gray). The parameters of the fitting trace are reported in Supplementary Table 6.

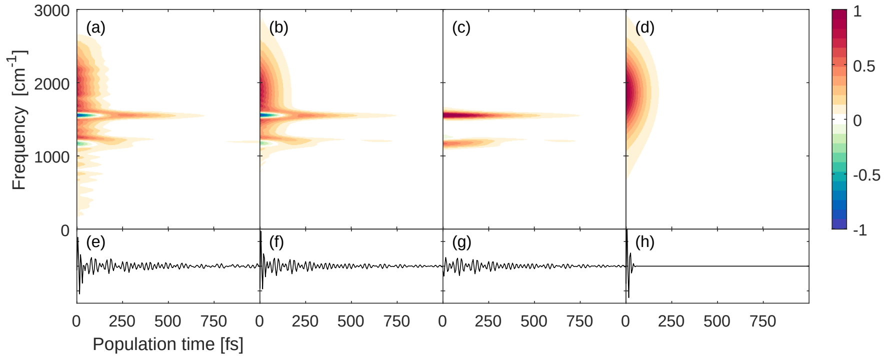

Supplementary Figure 9: Time-frequency analysis of S_2/Q_y cross-peak: experiment and simulation. Bilinear time-frequency transforms: (a) of the oscillating part of the experimental rephasing signal recorded with laser 3 at (17800,14850) cm^{-1} and reported in panel (e); (b) of the simulation of the aforementioned signal, shown in panel (f); (c) of the long-lasting oscillating components (panel (g)); and of the fast-dephasing beating (panel (h)).

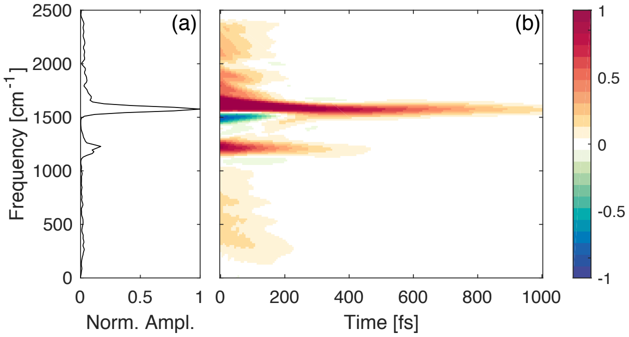

Supplementary Figure 10: Time-frequency analysis of Q_y/S_2 cross-peak. Fourier transform (a) and bilinear time-frequency transform (b) of the oscillating part of the rephasing signal recorded with laser 3 and extracted at coordinates corresponding to the Q_y/S_2 above diagonal cross-peak.

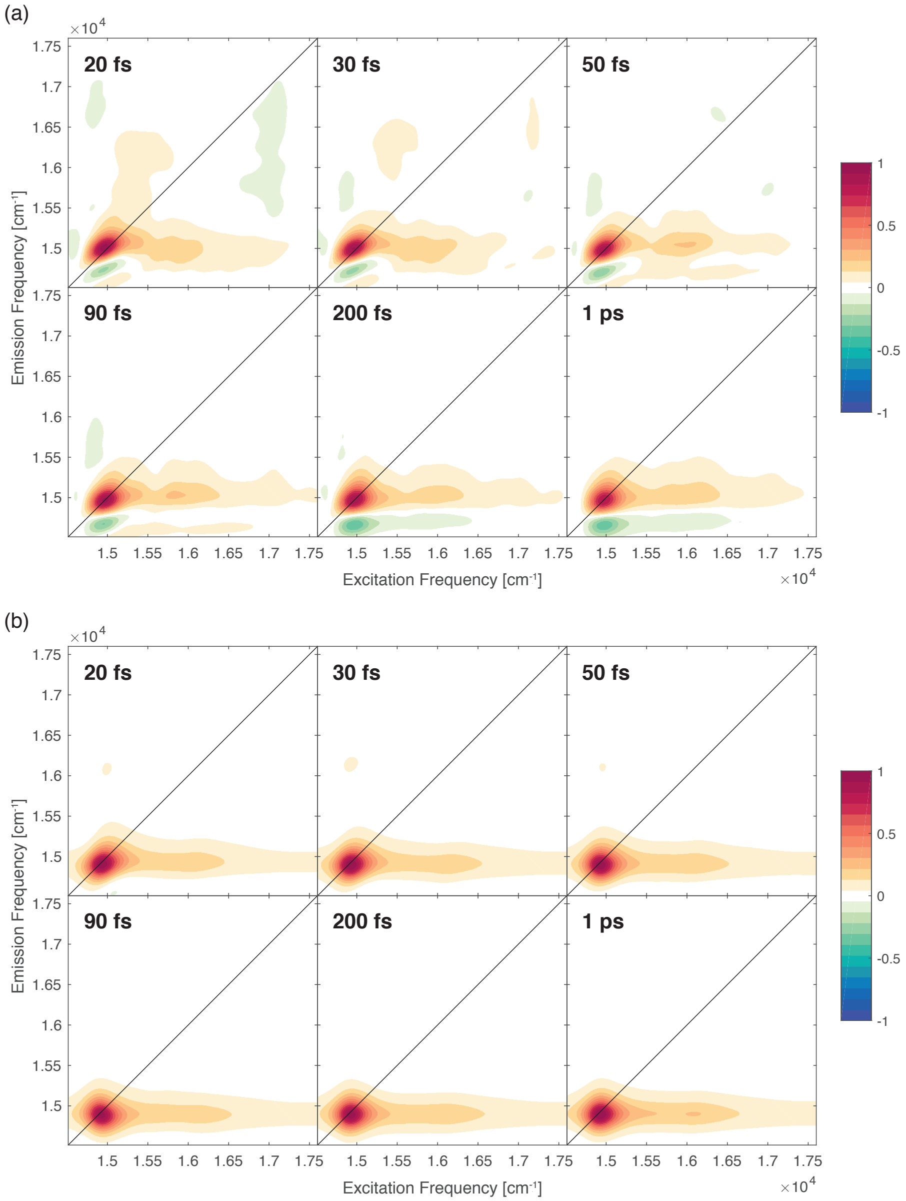

Supplementary Figure 11: 2DES maps recorded with laser 1. Evolution of pure absorptive 2DES maps along population time with laser 1. Experimental (a) and simulated (b) data.

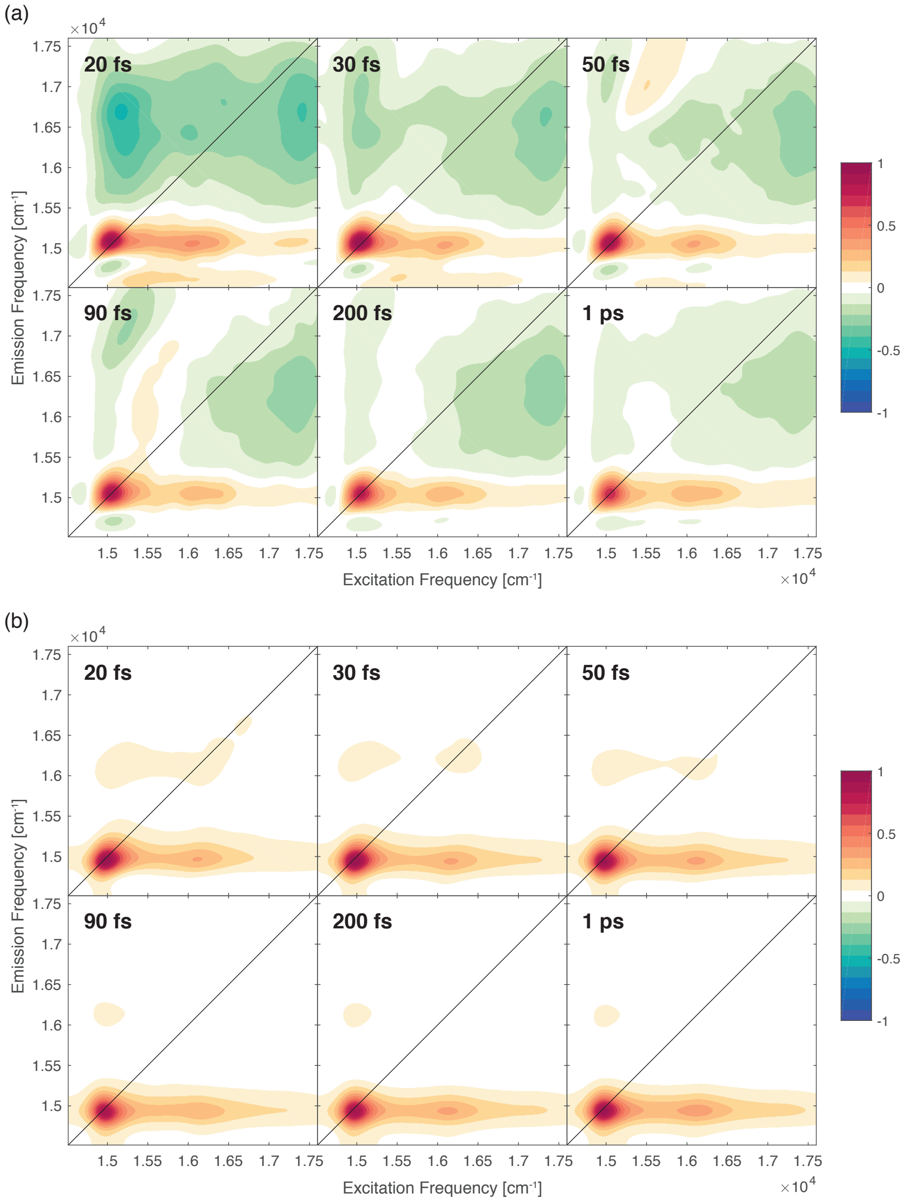

Supplementary Figure 12: 2DES maps recorded with laser 2. Evolution of pure absorptive 2DES maps along population time with laser 2. Experimental (a) and simulated (b) data.

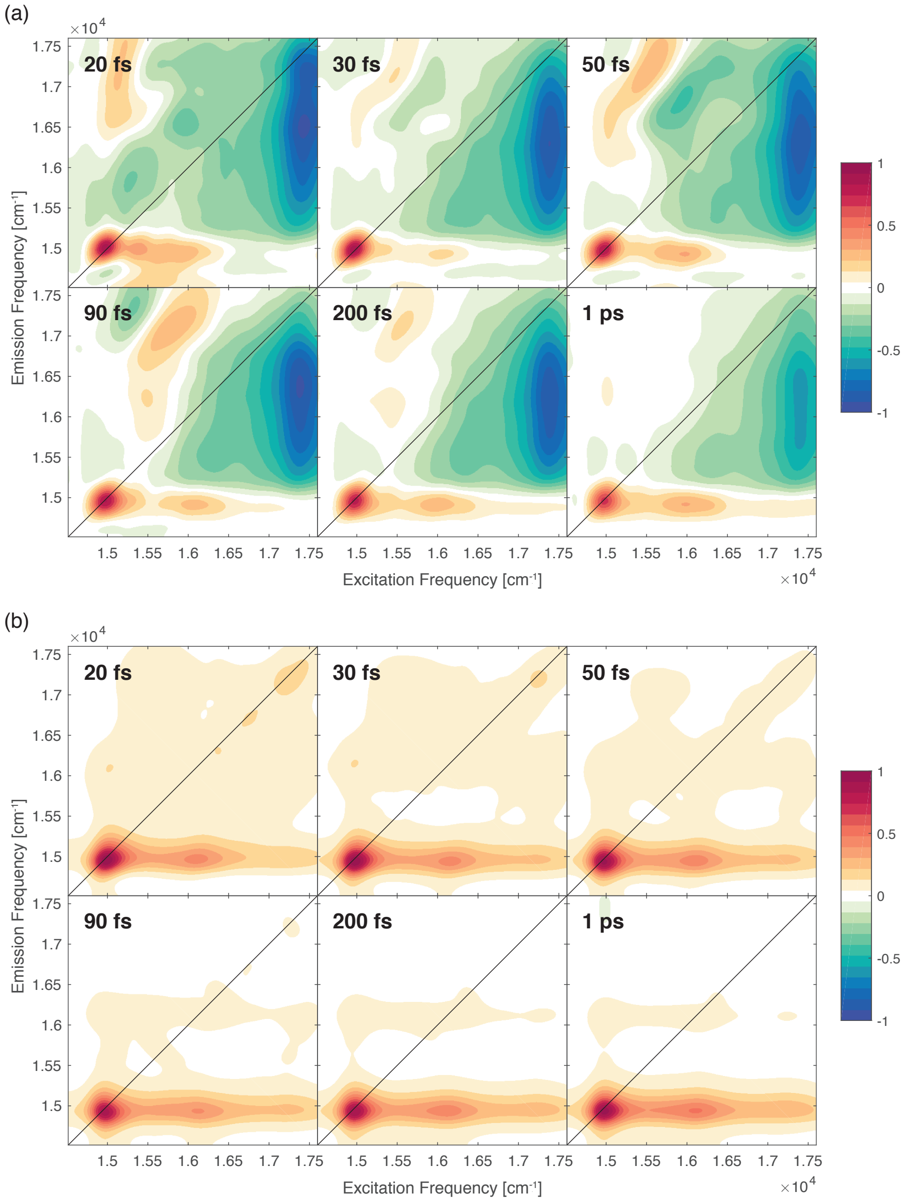

Supplementary Figure 13: 2DES maps recorded with laser 3. Evolution of pure absorptive 2DES maps along population time with laser 3. Experimental (a) and simulated (b) data.

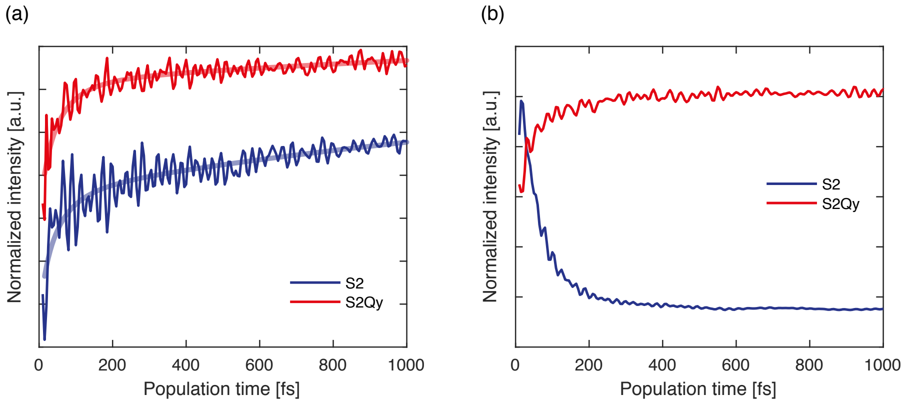

Supplementary Figure 14: Analysis of S_2/Q_y cross-peak. Evolution of the pure absorptive signal recorded with laser 3 along population time at selected excitation/emission energies. Experimental (a) and simulated (b) data extracted at coordinates corresponding to the dynamics of S_2 population (blue) and at the cross peak below diagonal corresponding to the S_2/Q_y transfer (red). Thick lines in panel (a) represent the fitting traces. The simulated data (blue trace in panel (b)) do not account for the $S_2 \rightarrow S_n$ ESA contribution.

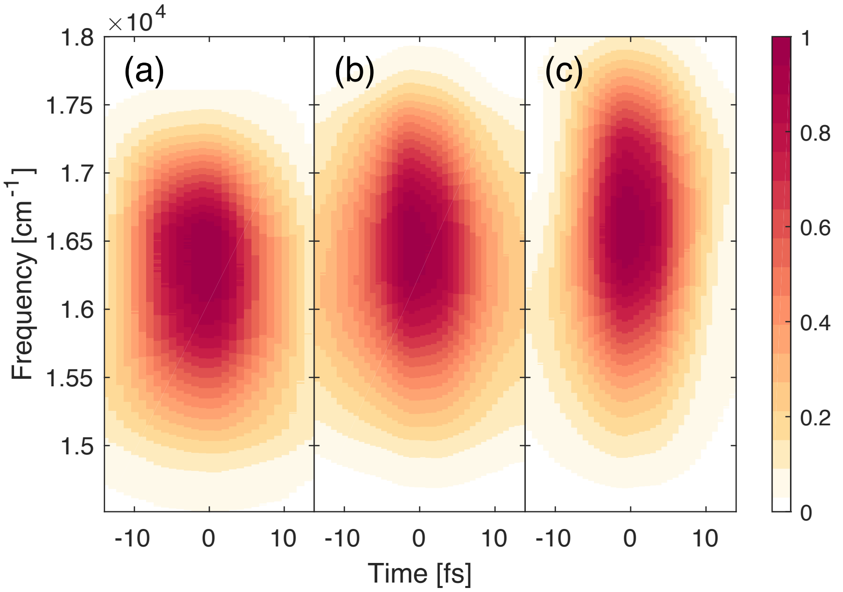

Supplementary Figure 15: FROG measurements. FROG measurement performed at sample position for laser 1 (a), laser 2 (b) and laser 3 (c).

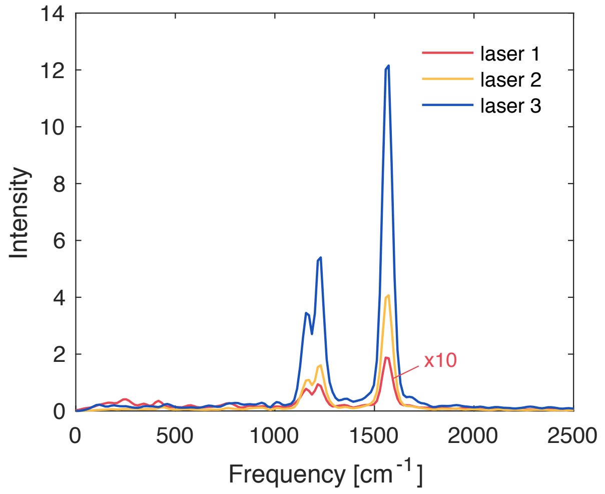

Supplementary Figure 16: Fourier transform analysis. Fourier transform of the oscillating part of the 2DES signal of the three datasets after subtraction of the noncoherent part of the signal determined by the kinetic global analysis. The full oscillating behavior of each dataset is obtained by integrating the Fourier spectra over excitation and emission axis. Intensity of laser 1 power spectrum is multiplied by a factor of 10. The three measures reveal the presence of three main signals at about 1160, 1230 and 1570 cm^{-1}, that match quite well the frequency of the three vibrational modes of Per more strongly coupled to the $S_0 \rightarrow S_2$ transition. The intensity of Per vibrational modes increases as the laser spectrum is shifted to higher energies, i.e. S_2 transition is progressively brought into resonance. Moreover, power spectrum recorded with laser 1 reveals several peaks below 1000 cm^{-1} due to Chl vibrational modes.

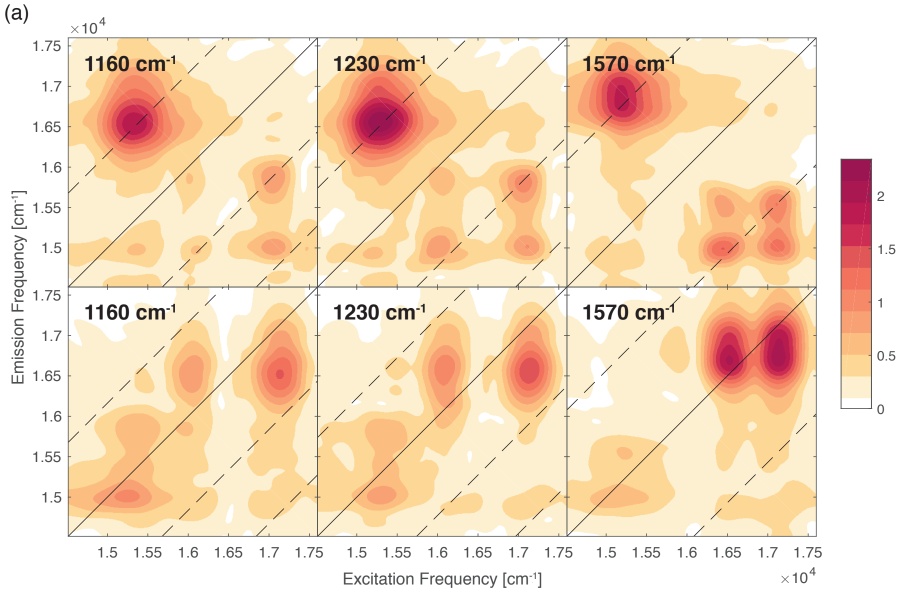

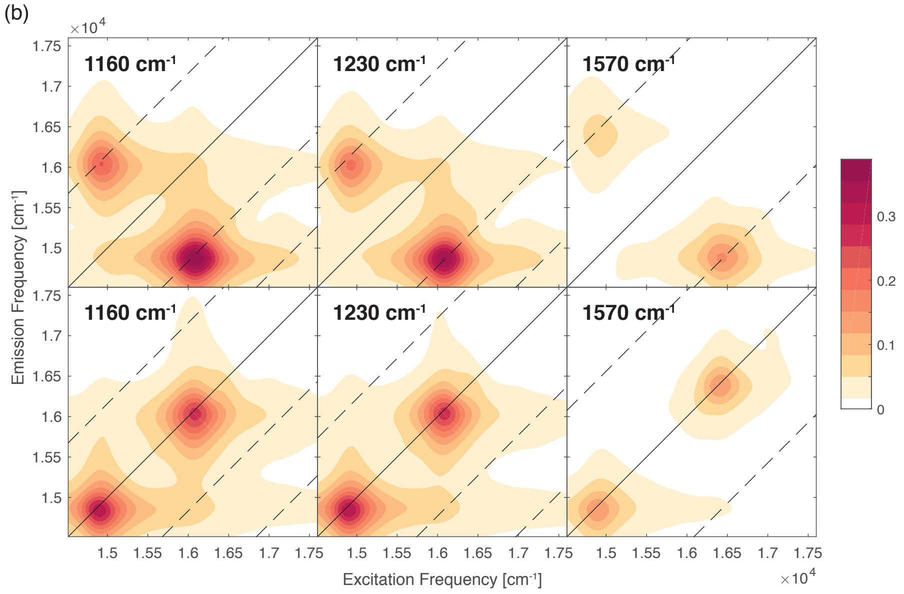

Supplementary Figure 17: Fourier maps for laser 1. Amplitude of the three main oscillating contributions as a function of excitation and emission frequency (Fourier maps) of the 2DES rephasing (upper row) and non-rephasing (lower row) datasets. Experimental data (a) and simulated data (b) obtained with laser 1. Experimental and simulated rephasing maps of all the vibrational modes differ mainly in the strong cross-peak above diagonal.

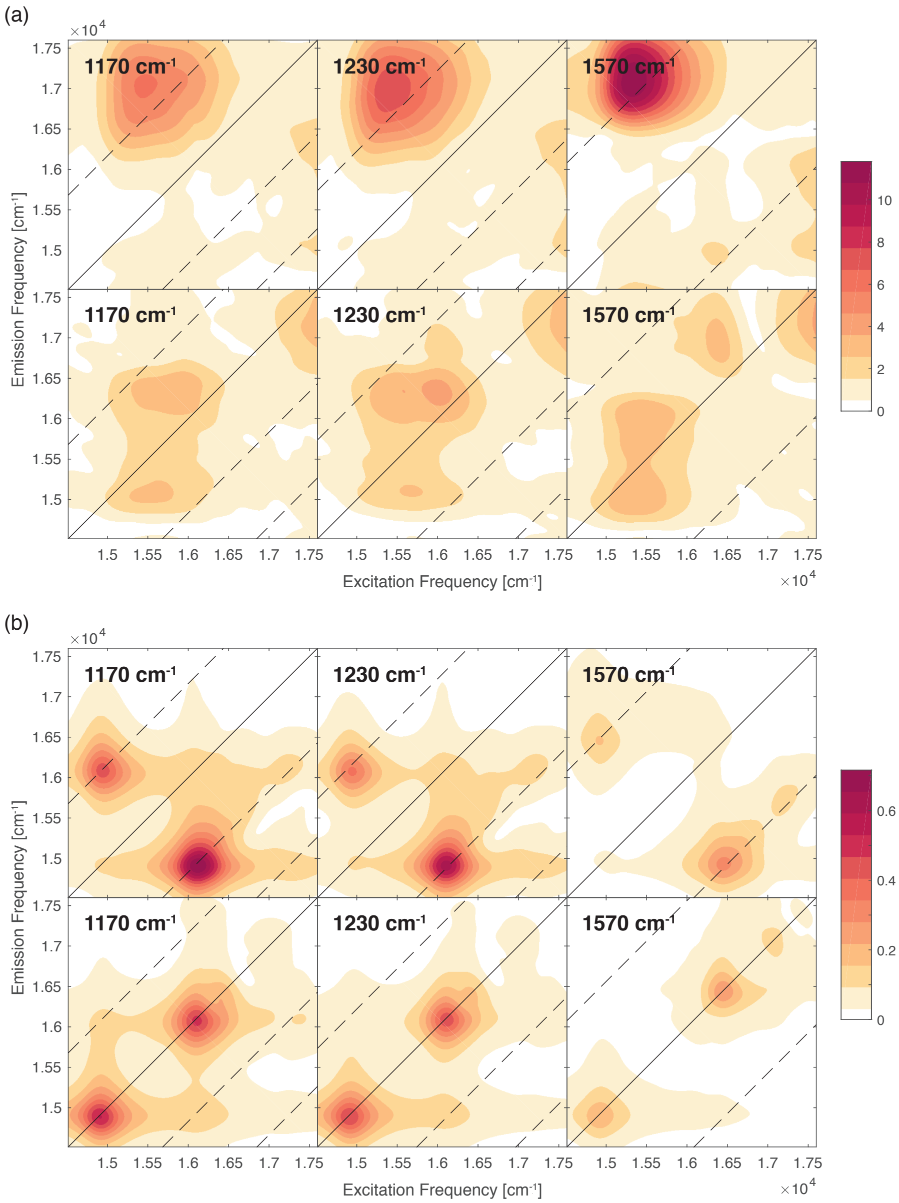

Supplementary Figure 18: Fourier maps for laser 2. Same as Supplementary Fig. 17 with laser 2. The intensity of the cross-peak above diagonal not predicted by simulations increases as the exciting pulses progressively bring S_2 transition into resonance.

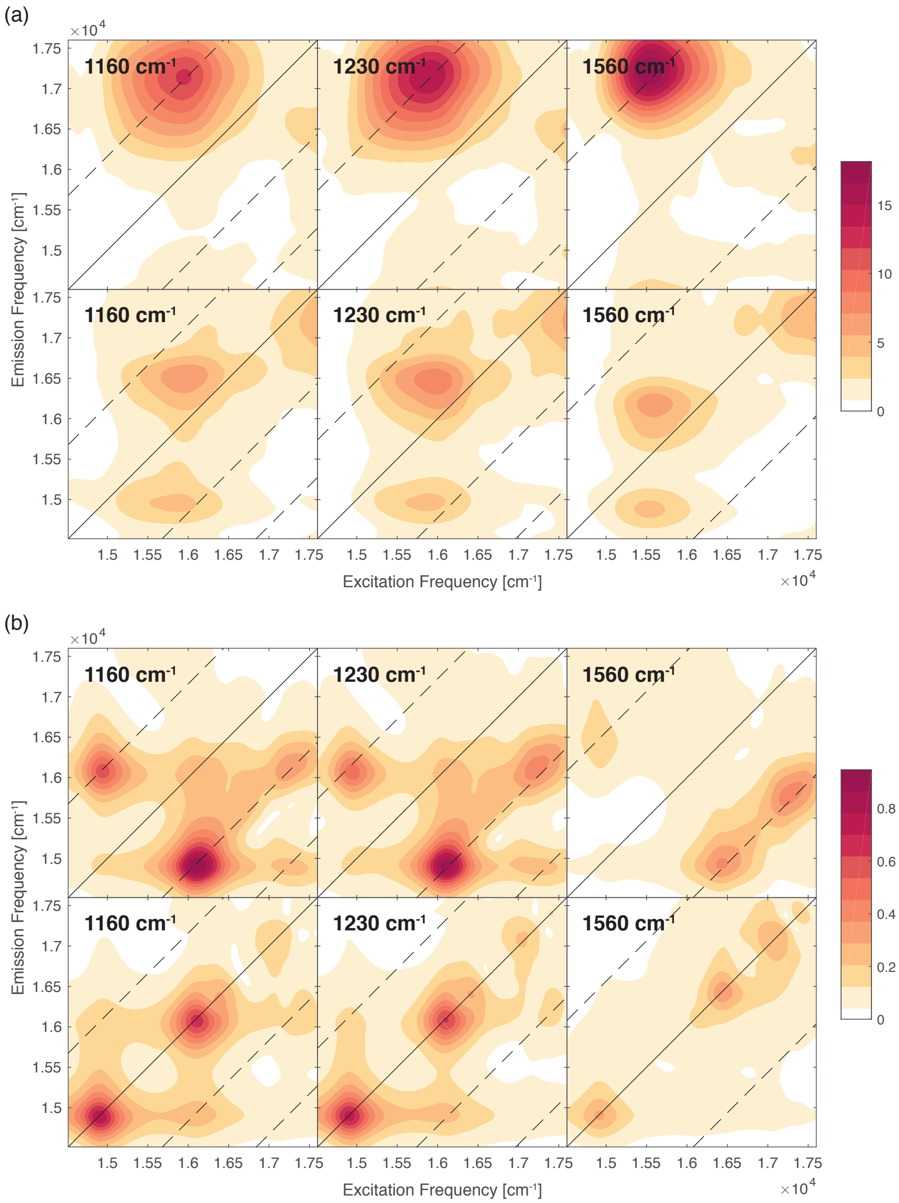

Supplementary Figure 19: Fourier maps for laser 3. Same as Supplementary Fig. 17 with laser 3. The intensity of the cross-peak above diagonal not predicted by simulations increases as the exciting pulses progressively bring S_2 transition into resonance.

Supplementary Table 1: Parameters of the static and dynamic disorder used in this work. Parameter λ_d is the (calculated) discrete contribution to the reorganization energy (second term of the rhs of Eq. S3), λ_{tot} is the total reorganization energy. The standard deviation of the static disorder on site energies is denoted as σ. All values are in cm^{-1}.

Pigment (Tr.)	σ	λ_d	λ_c	λ_{tot}
Chl601 (Q_y)	35	259	24	283
Chl602 (Q_y)	35	341	24	365
Chl601 (Q_x)	35	287	24	311
Chl602 (Q_x)	35	320	24	344
Chl (B_x/B_y)	35	648	36	685
Per (S_2)	800	1040	30	1070

Supplementary Table 2: Site energies for PCP pigments. We show the relevant excited states of the chlorophylls (top) and peridinins (bottom) in PCP. All values in cm^{-1}.

Chl	Q_y	Q_x	B_y	B_x
a601	16845	18364	24483	24968
a602	17018	18447	24320	24941

Per	611	612	613	614
	17051	17897	17373	17801
Per	621	622	623	624
	17155	17962	16849	16688

Supplementary Table 3: Couplings among the Q states of Chls 601/602. All values in cm^{-1}.

States		Coupling
601 Q_y	602 Q_y	-11.1
601 Q_y	602 Q_x	7.0
601 Q_x	602 Q_y	-6.8
601 Q_x	602 Q_x	-1.2

Supplementary Table 4: Couplings among the S_2 states of Pers in PCP. All values in cm^{-1}. Signs are consistent with an allene-lactone dipole direction in Per.

N-terminus		C-terminus	
Pers	Coupling	Pers	Coupling
611–612	445.9	621–622	368.2
611–613	203.8	621–623	197.1
611–614	218.5	621–624	217.7
612–613	212.0	622–623	219.7
612–614	122.4	622–624	121.3
613–614	363.3	623–624	378.4

Supplementary Table 5: Couplings between Pers and Chls. Most important couplings (in cm^{-1}) between Chls (Q_y/Q_x) and Pers in PCP. Signs are consistent with an allene-lactone dipole direction in Per.

Chl–Per pair	Coupling	
	Q_y–S_2	Q_x–S_2
601–611	241.4	27.4
601–612	144.5	-19.8
601–613	-136.3	-78.3
601–614	305.2	54.5
602–621	230.0	9.5
602–622	121.5	-24.8
602–623	-127.5	-72.1
602–624	308.4	35.9

Supplementary Table 6: Fitting parameters. Fitting parameters of the model function in Eq. S17.

Parameter	Value and standard error
τ_1 / fs	$(31 \pm 7) \cdot 10$
τ_2 / fs	$(83 \pm 26) \cdot 10$
τ_3 / fs	$(34 \pm 3) \cdot 10$
σ_4 / fs	16.8 ± 0.5
ω_1 / cm^{-1}	1152 ± 4
ω_2 / cm^{-1}	1214 ± 2.0
ω_3 / cm^{-1}	1551.0 ± 1.1
ω_4 / cm^{-1}	1872 ± 18

Supplementary References

[1] Lee, M. K., Huo, P. & Coker, D. F. Semiclassical path integral dynamics: Photosynthetic energy transfer with realistic environment interactions. *Annu. Rev. Phys. Chem.* **67**, 639–668 (2016).

[2] Zhang, W. M., Meier, T., Chernyak, V. & Mukamel, S. Exciton-migration and three-pulse femtosecond optical spectroscopies of photosynthetic antenna complexes. *J. Chem. Phys.* **108**, 7763–7774 (1998).

[3] Yang, M. & Fleming, G. R. Influence of phonons on exciton transfer dynamics: Comparison of the Redfield, Förster, and modified Redfield equations. *Chem. Phys.* **275**, 355–372 (2002).

[4] Novoderezhkin, V. I. & van Grondelle, R. Physical origins and models of energy transfer in photosynthetic light-harvesting. *Phys. Chem. Chem. Phys.* **12**, 7352–7365 (2010).

[5] Hofmann, E. *et al.* Structural Basis of Light Harvesting by Carotenoids: Peridinin-Chlorophyll-Protein from Amphidinium carterae. *Science* **272**, 1788–1791 (1996).

[6] Case, D. A. *et al.* Amber14, University of California, San Francisco. (2015).

[7] Vreven, T. *et al.* Combining quantum mechanics methods with molecular mechanics methods in ONIOM. *J. Chem. Theory Comput.* **2**, 815–826 (2006).

[8] Knecht, S., Marian, C. M., Kongsted, J. & Mennucci, B. On the photophysics of carotenoids: A multireference DFT study of peridinin. *J. Phys. Chem. B* **117**, 13808–13815 (2013).

[9] Hornak, V. *et al.* Comparison of multiple Amber force fields and development of improved protein backbone parameters. *Proteins Struct. Funct. Bioinforma.* **65**, 712–725 (2006).

[10] Curutchet, C. *et al.* Electronic energy transfer in condensed phase studied by a polarizable QM/MM model. *J. Chem. Theory Comput.* **5**, 1838–1848 (2009).

[11] Wang, J. *et al.* Development of polarizable models for molecular mechanical calculations I: Parameterization of atomic polarizability. *J. Phys. Chem. B* **115**, 3091–3099 (2011).

[12] Volpato, A., Bolzonello, L., Meneghin, E. & Collini, E. Global analysis of coherence and population dynamics in 2D electronic spectroscopy. *Opt. Express* **24**, 24773–24785 (2016).

[13] Abramavicius, D., Valkunas, L. & Mukamel, S. Transport and correlated fluctuations in the nonlinear optical response of excitons. *Europhys. Lett.* **80**, 17005 (2007).

[14] Mullen, K. M. & van Stokkum, I. H. M. The variable projection algorithm in time-resolved spectroscopy, microscopy and mass spectrometry applications. *Numer. Algorithms* **51**, 319–340 (2009).

[15] Kleima, F. J. *et al.* Peridinin Chlorophyll a Protein: Relating structure and steady-state spectroscopy. *Biochemistry* **39**, 5184–5195 (2000).

[16] Volpato, A. & Collini, E. Time-frequency methods for coherent spectroscopy. *Opt. Express* **23**, 20040–20050 (2015).
